# Supplementary material for: Using the Water and Sanitation for Health Facility Improvement Tool (WASH FIT) in Zimbabwe: A Cross-Sectional Study of Water, Sanitation and Hygiene Services in 50 COVID-19 Isolation Facilities
Source: Int J Environ Res Public Health. 2021 May 25;18(11):5641. doi: 10.3390/ijerph18115641 (PMC8197478; doi:10.3390/ijerph18115641)
Supplement: Supplementary file 1 [file ijerph-18-05641-s001.zip › ijerph-1221353-supplementary.pdf]

Table S1. WASH FIT indicator findings.

Indicator #	WASHFIT Indicators	Total	Small	Medium	Large
	**Water**				
1.1	Improved water supply piped into the facility or on premises and available	1.44	1.23	1.60	1.63
1.2	Water services available at all times and of sufficient quantity for all uses	0.94	0.91	0.85	1.25
1.3	A reliable drinking water station is present and accessible for staff, patients and carers at all times and in all locations/wards	1.12	1.00	1.05	1.63
1.4	Drinking water is safely stored in a clean bucket/tank with cover and tap	1.36	1.23	1.35	1.75
1.5	Sanitary inspection risk score	0.92	0.86	0.90	1.13
1.6	All endpoints are connected to an available and functioning water supply	1.10	0.95	1.00	1.75
1.7	Water services available throughout the year	1.30	1.18	1.25	1.75
1.8	Water storage is sufficient to meet the needs of the facility for two days	0.88	1.00	0.65	1.13
1.9	Water is treated and collected for drinking with a proven technology that meets WHO performance standards	1.24	1.23	1.20	1.38
1.10	Drinking water has appropriate chlorine residual or 0 *E.coli*/100 ml and is not turbid	1.04	0.82	1.15	1.38
1.11	The facility water supply is regulated according to national water quality standards	1.34	1.32	1.40	1.25
1.12	Energy is available for heating water	0.76	0.64	0.85	0.86
1.13	Energy is available for pumping water	1.04	0.95	0.95	1.50
1.14	At least one shower or bathing area is available per 40 patients in inpatient settings and is functioning and accessible	1.14	1.05	1.20	1.25
1.15	Shower(s) are adequately lit, including at night	1.06	1.05	1.00	1.25
	**Sanitation**				
2.1	Number of available and usable toilets or improved latrines for patients	1.04	1.09	0.95	1.13
2.2	Toilets or improved latrines clearly separaed for staff and patients	1.16	1.23	1.05	1.25
2.3	Toilets or improved latrines clearly separated for male and female	1.52	1.45	1.70	1.25
2.4	At least one toilet or improved latrine provides the means to manage menstrual hygiene needs	0.56	0.68	0.45	0.50
2.5	At least one toilet meets the needs of people with reduced mobility	0.34	0.18	0.50	0.38
2.6	Functioning hand hygiene stations within 5 m of latrines	1.14	1.09	1.10	1.38

2.7	Record of cleaning toilets visible and signed by the cleaners each day	0.88	0.86	0.90	0.88
2.8	Wastewater is safely managed through use of on-site treatment or sent to a functioning sewer system	1.22	1.14	1.20	1.50
2.9	Greywater drainage system is in place that diverts water away from the facility and also protects nearby households	1.36	1.18	1.50	1.50
2.10	Latrines are adequately lit, including at night	1.04	1.00	0.95	1.38
	**Health Care Waste**				
2.11	A trained person is responsible for the management of health care waste in the health care facility	1.36	1.55	1.20	1.25
2.12	Functional waste collection containers in close proximity to all waste generation points for: non-infectious (general) waste, infectious waste, and sharps waste	0.98	0.77	1.05	1.38
2.13	Waste correctly segregated at all waste generation points	1.04	0.95	1.05	1.25
2.14	Funcional burial pit/fenced waste dump or municipal pick-up available for disposal of non-infectious waste	1.46	1.45	1.35	1.75
2.15	Incinerator or alternative treatment technology for the treatment of infectious and sharp waste is functional and of a sufficient capacity	1.26	1.32	1.15	1.38
2.16	Sufficient energy available for incineration or alternative treatment technologies	0.88	0.76	0.72	0.99
2.17	Hazardous and non-hazardous waste are stored separately before being treated/disposed of or moved off site	1.20	1.55	1.00	0.75
2.18	All infectious waste is stored in a protected area before treatment for no longer than the default and safe time	1.12	1.14	1.05	1.25
2.19	Anatomical/pathological waste is put in a dedicated pathological waste/placenta pit, burnt in a crematory or buried in a cemetery	1.46	1.36	1.45	1.75
2.2	Dedicated ash pits available for disposal of incineration ash	1.16	1.18	1.05	1.38
2.21	Protocol or standard operating procedure for safe management of health care waste clearly visible and legible	1.56	1.50	1.45	2.00
2.22	Appropriate protective equipment for all staff in charge of waste treatment and disposal	0.80	0.82	0.75	0.88
	**Hand Hygiene**				
3.1	Functioning hand hygiene stations are available at all points of care	1.26	1.09	1.40	1.38

3.2	Handhygiene promotion materials clearly visible and understandable at key places	1.22	1.09	1.40	1.13
3.3	Functioning hand hygiene stations are available in service areas	1.18	1.05	1.35	1.13
3.4	Functioning hand hygiene stations available in waste disposal area	0.70	0.77	0.65	0.63
3.5	Hand hygiene compliance activities are undertaken regularly	1.48	1.32	1.70	1.38
	**Facility Environment, Cleanliness and Disinfection**				
3.6	The exterior of the facility is well-fenced, kept generally clean (free from solid waste, stagnant water, no animal and human feces in or around the facility premises)	1.50	1.50	1.45	1.63
3.7	General lighting sufficiently powered and adequate to ensure safe provision of health care including at night	1.20	1.23	1.15	1.25
3.8	Floors and horizontal work surfaces appear clean	1.42	1.23	1.70	1.25
3.9	Appropriate and well maintained materials for cleaning are available	1.06	0.86	1.15	1.38
3.10	At least two pairs of household cleaning gloves and one pair of overalls or apron and boots in a good state for each cleaning and waste disposal staff member	0.88	0.77	1.05	0.75
3.11	At least one member of staff can demonstrate the correct procedures for cleaning and disinfection and apply them as required to maintain clean and safe rooms	1.46	1.45	1.50	1.38
3.12	Beds have insecticide treated nets to protect patients from mosquito-borne diseases	0.72	0.50	1.00	0.63
3.13	A mechanism exists to track supply of IPC-related materials to identify stock-outs	1.48	1.55	1.30	1.75
3.14	Record of cleaning visible and signed by the cleaners each day	0.50	0.41	0.70	0.25
3.15	Laundry facilities are available to wash linen from patients beds between each patient	1.32	1.14	1.35	1.75
3.16	The facility has sufficient natural ventilation and where the climate allows, large opening windows, skylights and other vents to optimize natural ventilation	1.80	1.73	1.90	1.75
3.17	Kitchen stores and prepared food is protected from flies, other insects or rats	1.02	1.23	0.80	1.00
3.18	Beds for patients should be separated by 2.5 m from the center of one bed to th enext and each bed has only one patient	1.78	1.73	1.80	1.88
	**Management**				

4.1	WASH FIT or other quality improvement/management plan for the facility is in place, implemented and regularly monitored	0.94	0.86	1.05	0.88
4.2	An annual planned budget for the facility is available and includes funding for WASH infrastructure, services, personnel and the continuous procurement of WASH items which is sufficient to meet th eneeds of the facility	0.72	0.50	0.75	1.25
4.3	An up-to-date diagram of the facility management structure is clearly visible and legible	1.30	1.05	1.45	1.63
4.4	Adequate cleaners and WASH maintenance staff are available	1.14	1.14	1.15	1.13
4.5	A protocol for operation and maintenance, including procurement of WASH supplies is visible, legible and implemented	1.32	1.27	1.25	1.63
4.6	Regular ward-based audits are undertaken to assess the availability of handrub, soap, single use towels and other hand hygiene resources	1.24	1.41	1.05	1.25
4.7	New health care personnel receive IPC training as part of their orientation programme	1.08	1.18	1.00	1.00
4.8	Health care staff are trained on WASH/IPC each year	1.02	1.05	1.00	1.00
4.9	Facility has a dedicated WASH or IPC focal person	1.48	1.45	1.50	1.50
4.10	All staff have a job description written clearly and legibly, including WASH-related responsibilities and are regularly appraised on their performance	1.74	1.59	1.85	1.88
4.11	High performing staff are recognized and rewarded and those that do not perform are dealt with accordingly	0.72	0.45	0.75	1.38
